# Therapeutic Potential of the Microbiome in the Treatment of Neuropsychiatric Disorders

**DOI:** 10.3390/medsci7020021

**Published:** 2019-01-31

**Authors:** Alper Evrensel, Barış Önen Ünsalver, Mehmet Emin Ceylan

**Affiliations:** 1 Department of Psychiatry, Uskudar University, PK:34768 Istanbul, Turkey; 2 NP Brain Hospital, Saray Mahallesi, Ahmet Tevfik İleri Cad. No: 18, PK:34768 Umraniye Istanbul, Turkey; 3 Vocational School of Health Services, Department of Medical Documentation and Secretariat, Uskudar University, PK:34768 Istanbul, Turkey; onenunsalver@gmail.com; 4 Departments of Psychology and Philosophy, Uskudar University, PK:34768 Istanbul, Turkey; m.eminceylan@yahoo.com

**Keywords:** gut microbiota, microbiome, dysbiosis, psychiatry, neurology, fecomodulation, fecal microbiota transplantation, probiotics, prebiotics

## Abstract

The search for rational treatment of neuropsychiatric disorders began with the discovery of chlorpromazine in 1951 and continues to evolve. Day by day, new details of the intestinal microbiota–brain axis are coming to light. As the role of microbiota in the etiopathogenesis of neuropsychiatric disorders is more clearly understood, microbiota-based (or as we propose, “fecomodulation”) treatment options are increasingly discussed in the context of treatment. Although their history dates back to ancient times, the importance of psychobiotics and fecal microbiota transplantation (FMT) has only recently been recognized. Despite there being few preclinical and clinical studies, the evidence gathered to this point suggests that consideration of the microbiome in the treatment of neuropsychiatric disorders represents an area of significant therapeutic potential. It is increasingly hoped that such treatment options will be more reliable in terms of their side effects, cost, and ease of implementation. However, there remains much to be researched. Questions will be answered through germ-free animal experiments and randomized controlled trials. In this article, the therapeutic potential of microbiota-based options in the treatment of neuropsychiatric disorders is discussed in light of recent research.

## 1. Introduction

Scientific consensus dates the birth of modern psychopharmacology back to 1951 [1]. Similar to the discovery of penicillin, the serendipitous discovery and synthesis of chlorpromazine opened the gates of psychopharmacology and synaptic neurotransmission [2]. The discovery of psychotropics that followed led to further developments in the field [3]. As a consequence of thousands of studies, enormous steps have been taken in terms of the etiopathogenesis and treatment of neuropsychiatric disorders. 

Beginning in the 2000s, the focus of psychiatry began to shift from synaptic transmission and monoamine receptor functions to areas such as intestinal microbiota [4], the peripheral system [5], mitochondria [6,7], the mitochondria–microbiota interaction [8], the immune system [9], and neuroinflammation [10]. 

One of these new areas of interest is the microbiota. Prokaryotes were the first living organisms on earth, and quantitively are the largest group amongst extant species [11]. Humans constitute a “super organism” comprising a combination of eukaryotic and prokaryotic cells [12]. Microorganisms have colonized almost every region of the body [13]. These guests communicate with human body cells (just as they do amongst themselves) [14] in a cooperative and commensal manner [15]. It has been suggested that the brain is not sterile and that a “brain microbiota” may also exist [16,17]. 

The intestinal microbiota is composed of commensal prokaryotes which colonize the intestines (especially the large intestine) [18]. It is estimated that there are approximately 380 trillion microbes in the intestinal microbiota. This is approximately four times the number of adult human cells [19]. The genome of the prokaryote microbiota is large, containing more genes than that of a human [20]. Included in this microbiota are commensal fungi (a mycobiome) [21]. 

Three-quarters of the microbiota in an adult human is composed of *Firmicutes* and *Bacteroidetes*. The ratio of *Firmicutes/Bacteroidetes* is estimated to be approximately 11% [22]. Microbiota composition is dynamic and unique and can vary depending on the host’s genetic structure, age, nutrition, stress level, medicinal intake, and place of residence (rural or urban) [23,24,25]. For example, olanzapine (an atypical antipsychotic drug) increases *Firmicutes* levels and reduces *Protobacteria* and *Actinobacteria* levels [26]. *Bifidobacteria* and *Lactobacilli* levels decrease in newborns exposed to stress [27,28]. Chronic stress reduces the proportion of *Bacteroides* and increases the proportion of *Clostridium* species in the host microbiome [29]. Antibiotics may trigger cytokine imbalances in patients with depression [30]. Use of antibiotics before the age of one correlates with adulthood depression [31]. Long-term broad-spectrum antibiotic use can permanently alter the composition of the intestinal microbiota [32]. Interestingly, minocycline produces antipsychotic-like effects [33].

Elie Metchnikoff’s Nobel Prize-winning work in 1908 was related to the role of probiotic microorganisms in the immune system and human health [34]. Shortly after this, the first article to report on the success of *Lactobacillus* in the treatment of depression (“melancholy” as stated in the article) was published [35]. The idea that there is a correlation between the occurrence of allergies and the use of antibiotics, urban environments, and increased use of cleaning products is known as the “hygiene hypothesis” [36]. Another hypothesis suggests that “old friend” microorganisms have very important functions in human evolution [37,38]. These views have formed the basis of our current understanding of human–microbe interactions.

The study of the intestinal microbiota–brain axis is progressing rapidly today. There is evidence that the intestinal microbiota can act on neuronal function by both direct and indirect means [18]. The immune system seems to play a key role in this bidirectional interaction [39]. It is now thought that both neurons and the brain are affected by many intrinsic and extrinsic factors and that microbiota-induced neuroinflammation has an important role in the etiopathogenesis of neuropsychiatric disorders [40].

Seminal discoveries in the field have led to the realization that the intestinal microbiota–immune system–brain axis must be considered in the treatment of neuropsychiatric disorders. This approach, referred to as “microbiota-based therapy” includes prebiotics, probiotics (psychobiotics), and fecal microbiota transplantation (FMT). In the scientific literature, there are numerous reviews of the mechanisms underlying the gut–brain relationship. However, there are few articles on the therapeutic potential of microbiota in the treatment of neuropsychiatric disorders. In articles related to microbiota-based therapies, psychobiotics are more commonly emphasized. We found very few articles on the potential use of FMT in neuropsychiatric disorders. Therefore, in this article, we propose the term “fecomodulation” and discuss in detail the neuropsychiatric therapeutic potential of these treatments in light of the current literature. 

## 2. The Microbiome as a New Psychiatric Treatment Option

The incidence and prevalence of psychiatric diseases have increased in the last three–four decades [41,42]. One in every 40 children in the United States is diagnosed with autism spectrum disorder [43]. It has been suggested that this rapid increase may be due to modern dietary habits such as frequent consumption of fast food [44]. Rates of food-related disorders can be reduced through dietary regulation [45]. In addition, the importance of prebiotics and probiotics (psychobiotics) is increasingly recognized [39]. In order to understand approaches for which the aim is to manipulate intestinal microbiota to achieve positive therapeutic outcomes, we will first discuss in detail the gut–brain relationship. 

### 2.1. The Gut-Brain Axis

Our body is a complex system in which our own cells and commensal microbes coexist symbiotically [40]. Bacteria that live in the intestine and have positive effects on neuronal function are called “psychobiotics” [46]. Psychobiotics play an important role in the immune system, endocrine system, and metabolism by secreting neurohormones, neurotransmitters, and neuropeptides. In this way, they have an important role in the body, especially with regard to brain function [4]. This bidirectional interaction between the intestines and the brain is called the “gut–brain axis” [18]. 

The digestive tract epithelium has a surface area the size of a tennis court and is the largest mucosal surface in the human body. In normal mucosa, enterocytes are tightly fixed to each other by tight junctions. Enterocytes also secrete mucus. Mucus prevents toxins from directly contacting enterocytes. This effective barrier can be impaired for various reasons (e.g., stress, exogenous glucocorticoids, dysbiosis, and endotoxins) and a “leaky gut” may result. At this stage, bacteria and their toxic metabolites (lipopolysaccharides) can interfere with blood and trigger inflammation [47,48]. Neuroinflammatory parameters increase in autistic mice [49,50]. Psychobiotics reduce the severity of symptoms associated with a leaky gut and neuroinflammation [48,51,52]. For example, *Bacteroides fragilis* can repair bowel leakage and reduce endotoxin-induced autistic behavior [53]. Anti-inflammatory activities of psychobiotics are important in neuropsychiatric disorders (e.g., depression) with low-grade neuroinflammation [4]. However, it is thought that psychobiotics have other beneficial effects as well [54,55].

Microbes can cooperate with the immune system. Prokaryotic cell elements (e.g., cell wall proteins and nucleic acids) activate immune system cells [18]. This contact is established via pattern recognition receptors (PRRs) and toll-like receptors (TLRs) [4]. Commensal bacteria can increase the level of interleukin-10 (IL-10, an anti-inflammatory cytokine) through activation of PRRs [56]. Probiotics such as *Lactobacillus* GG and *Bifidobacterium infantis* increase IL-10 levels, reduce proinflammatory cytokine levels, and reduce blood–brain barrier permeability [57]. Psychobiotics inhibit the proinflammatory process by stimulating TLR-2 and TLR-4 [58]. 

The brain has its own lymphatic drainage system [59]. This lymphatic system forms the basis of the interaction between neurons and the immune system. An increase in proinflammatory cytokines due to pathogenic microbes can alter brain levels of neurotransmitters [60]. They may also provoke proinflammatory processes by inducing prostaglandin synthesis [61]. Psychobiotics can reduce the level of proinflammatory cytokines in systemic circulation and low-level neuroinflammation. However, in a mouse trial, *Bifidobacterium longum* NCC3001 reduced anxiety levels and normalized low hippocampal brain-derived neurotrophic factor (BDNF) levels without any change in cytokine levels. These findings suggest that probiotics may also act through non-cytokine mechanisms, which could explain the results of the *Bifidobacterium longum* trial [62]. Brain-derived neurotrophic factor and N-methyl D-aspartate (NMDA) 2a levels in the hippocampus of germ-free (GF) mice are lower than those in mice with a diverse microbiota [63]. *Lactobacillus helveticus* and *Bifidobacterium longum* reduced anxiety scores in an animal experiment [54] and the same bacteria had similar effects on healthy human volunteers [64]. 

Another means of contact between the microbiota and nervous system is the myenteric plexus. Myenteric neurons are in the submucosa and establish direct contact with microorganisms in the lumen [65]. *Bifidobacterium longum* shows its anxiolytic effect by impacting myenteric neurons [66]. *Lactobacillus reuteri* reduces neuronal hyperexcitation [67]. Probiotics change peristaltism by affecting ion transport [68]. Microbiota bacteria are also effective in modifying the function of glia cells in addition to neurons [69]. In addition to these effects on bacteria, GF conditions also have unique effects on neuron functioning. Several abnormalities have been detected in the enteric nervous system of GF mice [70] and the neuronal excitability levels of such mice are low [71].

One of the most important means of communication within the gut–brain axis is the vagus nerve. The vagus nerve regulates parasympathetic activity between the brain and gastrointestinal tract and has effects on immune system function [72]. Stress, nutrition, and exercise affect vagal nerve tone [73,74,75]. Stimulation of the vagus nerve produces analgesic [76], anti-inflammatory [72], antidepressant-anxiolytic [77], and antiepileptic [78] effects. Antidepressant and anxiolytic drugs also affect vagal tone [79]. While some studies reported psychobiotic activity after vagotomy [66,80], no such activity was reported in another study [81]. However, it appears that the vagus nerve has an important function in psychobiotic activity. 

Dietary-derived plant fiber is digested with enzymes synthesized by the microbiota [82]. Short-chain fatty acids (SCFAs) are produced from fiber in the intestinal lumen [83]. Short-chain fatty acids (acetate, butyrate, propionate, and lactate) may cross the blood–brain barrier, but their effect on synaptic transmission is still unclear [84]. By affecting free fatty acid receptors [85] and through epigenetic mechanisms [86], SCFAs can alter neuromodulation [87]. 

Bacteria of the microbiota have metabolic functions other than SCFA production. These metabolites have an important role in the development of neuroinflammation [88]. Plasma tryptophan levels of GF mice are higher than in normal (i.e., with microbiota) mice. The plasma serotonin levels of normal mice are approximately three-fold higher than in GF mice [89]. Brain-derived neurotrophic factor levels, 5-hydroxitriptamine (serotonin) receptor 1A (5HT1A) expression [80], and anxiety scores are low in GF mice [90]. Bacteria of the microbiota may also have an effect on the metabolism of enterochromaffin cells. 

Intestinal bacteria synthesize neurotransmitters. *Escherichia* produce noradrenaline and serotonin, *Enterococcus* and *Streptococcus* produce serotonin, *Bacillus* produce dopamine and noradrenaline, and *Bifidobacteria* and *Lactobacillus* produce γ-aminobutyric acid (GABA) [91]. These neurotransmitters may affect the activity of the enteric nervous system. The microbiota also have effects on brain function by producing neuroactive substances (e.g., synaptophysin, BDNF, post-synaptic density-95) [92]. Studies on this subject are shown in Table 1. 

### 2.2. Therapeutic Potential of Psychobiotics

Unexpected results from animal experiments have revealed that the microbiome may have potential in the treatment of neuropsychiatric disorders. In this respect, probably the first article on the efficacy of *Lactobacilli* in the treatment of depression was published in 1910 [35]. In recent years (and because of increasing interest in the subject) many animal trials have been performed. A study on the psychobiotic activity of *Bifidobacterium infantis* reported that although there was no significant difference between the test protocols of the experimental and control groups, there was evidence of antidepressant-like activity in blood tests [93]. There was no significant difference between two groups in a study in which the antidepressant activity of *Bifidobacterium infantis* was compared with citalopram (an antidepressant) [94]. *Bifidobacterium pseudocatenulatum* CECT 7765 showed anxiolytic activity by reducing the acute stress response in mice. Additionally, inflammatory markers decreased and dysbiosis regressed [95]. *Lactobacillus rhamnosus* JB-1 has an anxiolytic effect by modulating GABA (an inhibitory neurotransmitter) function [96]. In contrast to these findings, the same bacterium was found to increase the level of glutamate (an excitatory neurotransmitter) [97]. Anxiolytic activity of another *Lactobacillus* strain (*Lactobacillus helveticus*) was compared with citalopram and found to be similar [98]. In another recent study on the psychobiotic efficacy of *Lactobacilli*, the antidepressant and anxiolytic activity of *Lactobacillus rhamnosus JB-1* was compared with fluoxetine (an antidepressant). Psychobiotic activity was observed in BALB/c mice, but not observed in Swiss Webster mice [99]. 

There are few human studies on the effectiveness of psychobiotics in various neuropsychiatric conditions. In a study comparing *Lactobacillus salivarius* and *Bifidobacterium infantis* in subjects with irritable bowel syndrome (48 females, 27 males), the positive effects observed with *Bifidobacterium* were not found in the group receiving *Lactobacillus* [57]. Two years later, in a trial using *Lactobacillus casei* Shirota, a partial positive effect on mood and negative effects on cognitive function were found [100]. In two randomized control trials published in 2011, the efficacy of *Lactobacillus helveticus R0052* and *Bifidobacterium longum* on neuropsychiatric parameters was evaluated. While depression and anxiety scores regressed, no significant change was found in tests that measured cognitive function [54,64]. In another study, *Bifidobacterium longum* 1714 use increased cognitive performance [101]. In a more recent study, high *Bacteroides* levels and low *Lachnospiraceae* levels were found in the stool of patients diagnosed with depression [102].

In a study assessing the effectiveness of psychobiotics on performance anxiety, *Lactobacillus casei* strain Shirota that was used for eight weeks decreased plasma cortisol levels before an exam [103], while in the other, *Lactobacillus gasseri* reduced the fatigue level of athletes after a competition [104]. Positive results were obtained in two studies that analyzed the psychobiotic activity of *Lactobacillus* and *Bifidobacteria* with functional magnetic resonance imaging fMRI [105,106]. In spite of these positive results, negative results have been reported in some studies [100,107]. These conflicting results may arise from methodological differences. Therefore, randomized controlled studies with large samples are needed. 

Bacteria are living entities that require nutrients to live. Our food is the source of their nutrients. Nutrients of probiotic bacteria are called “prebiotics” [18]. For example, *Bacteroides fragilis* and *Faecalibacterium prausnitzii* synthesize SCFAs by digesting fiber [108,109]. Therefore, fiber has the characteristics of a prebiotic.

Psychotropic drugs used in the treatment of neuropsychiatric disorders may alter the composition of the microbiota. Most psychotropic drugs show antibiotic activity [25]. The first antidepressant molecule was iproniazid (a monoamine oxidase (MAO) inhibitor), an anti-tuberculosis drug [110]. The positive effects of psychobiotics can be negated when they are administered to patients receiving psychotropic agents. The potent antipsychotic chlorpromazine has antibiotic/antifungal activity [111]. Monoamine oxidase inhibitors produce an antimicrobial effect by inhibiting cell wall synthesis, selective serotonin reuptake inhibitors (SSRIs) by inhibiting the efflux pump, and tricyclic antidepressants show an antimicrobial effect by inhibiting DNA gyrase (an antiplasmid effect) [25]. In addition to their synaptic effects, currently prescribed psychotropics may also contribute to a therapeutic effect by altering the composition of the microbiota [4].

### 2.3. Therapeutic Potential of Fecal Microbiota Transplantation

The fecal microbiota transplantation is a treatment method that dramatically alters the composition of the intestinal microbiota and correct dysbiosis [112]. In order to recognize the potency of FMT in the treatment of neuropsychiatric disorders, it is useful to look at the research in which fecal microbiota analysis has been performed. 

In a study [113], the composition of bacteria in the stool of patients with depression (*n* = 46) and healthy individuals (*n* = 30) was compared. *Firmicutes* levels were found to be lower in the stool of patients with depression, while the levels of *Bacterioides, Proteobacteria,* and *Actinobacteria* were found to be higher in the group with depression [113]. The authors interpreted this finding as being the consequence of patients with depression having a decreased proportion of beneficial bacteria as compared to harmful bacteria. First-episode psychosis cases (*n* = 28) and healthy individuals (*n* = 16) were compared in a more recent study in which the fecal microbiota composition was determined using quantitative real-time PCR and metagenomic analyses [114]. The stool of the participants were analyzed at the beginning and at the 2nd and 12th months. Fecal *Lactobacillus* levels of first-episode psychosis patients increased with treatment and this increase was correlated with symptom severity and treatment response. Even though the results of these studies are significant, further research is needed if stool microbiota analysis is to be used as a biomarker and to establish a cause–effect relationship. 

An experiment by Zheng et al. is considered seminal in this field. In their GF mouse experiment, mice transplanted with a “depression microbiota” taken from patients with depression were found to have a pattern of behavior consistent with depression, as compared to mice transplanted with a “healthy microbiota” taken from healthy individuals [115]. Kelly and colleagues achieved similar results in another study of similar design entitled “Transferring the Blues” [116]. 

There are case reports that FMT reduces the symptoms of autism in children [117]. In a study performed on a small sample (*n* = 18), children with autism underwent FMT (microbiota transfer therapy) following antibiotic (vancomycin) therapy for two weeks to modify intestinal microbiota [118]. The behavioral and gastrointestinal symptoms of autism decreased, and this condition lasted for 8 weeks. Fecal microbiota transplantation that is done once might not be sufficient to restore the microbiota composition sufficiently. Residual pathogenic bacteria (pathobionts) might be proliferating in time, resulting in the formation of old microbiota composition. Repetitive FMT may be needed to keep the restored and healthy microbiota. However, experimental and clinical studies are needed to further clarify these issues. 

The main indications for FMT are *Clostridium difficile* infection (CDI) and inflammatory bowel disease (ulcerative colitis and Crohn’s disease). Fecal microbiota transplantation is considered to be a reliable treatment method for these conditions, as the health of the microbiota in the stool of these patients is quite poor. In a follow-up study, serious side effects were not reported [119]. Patients may experience abdominal pain, gas, diarrhea, and constipation on the day of FMT application [120]. In a study with a large sample (*n* = 317), complications (bleeding, peritonitis, and enteritis) were reported only in three cases [121]. In another study, colitis was exacerbated [122]. Serious complications such as death due to peritonitis can also be seen following FMT that was done for conditions other than neuropsychiatric disorders [123]. The risks of FMT in psychiatric indications are not yet known. Randomized controlled trials are needed to clarify these risks. 

## 3. Conclusions

The philosopher of science Thomas Samuel Kuhn defined the concept of “paradigm shift” in his book *The Structure of Scientific Revolutions* [124]. Evidence presented in recent years indicates that modern psychopharmacology has been undergoing a significant paradigm shift in the last 70 years, resulting in significant progress in the study of the pathophysiology and treatment of neuropsychiatric disorders. Although the mechanisms have not yet been elucidated, the intestinal microbiota appear to play a role in the development of neuropsychiatric disorders. The research to date suggests that microbiota-based treatment can positively affect synaptic functioning. Although there exists some promising evidence for this, there are still many questions to be answered. Numerous randomized controlled trials are required to obtain these answers.

## Figures and Tables

**Table 1:** Summary of important preclinical and clinical studies on the gut–microbiota–brain axis.

Target Sample	Evaluation Tools	Outcomes and Mechanism of Disease Modulation	Research
**11 healthy subjects**	Fecal sample analysis via 16S rRNA	Microbiota composition may change based on diet.	David et al. [23]
**Wistar rats**	Feeding patterns, food preference, locomotor activity, and body temperature	Olanzapine leads to increases in the levels of *Firmicutes* and decreases in the levels of *Protobacteria* and *Actinobacteria*.	Davey et al. [26]
**Male Sprague Dawley rat pups**	Endocrine and immune measurements, polymerase chain reaction of partial 16S ribosomal RNA gene fragments and analysis by denaturing gradient gel electrophoresis	The stress caused by separation from the mother reduces the levels of *Bifidobacteria* and *Lactobacilli* in newborn rat guts.	O’Mahony et al. [28]
**Male CD-1 mice**	Social disruption tests, real-time PCR, bacterial tag-encoded FLX amplicon pyrosequencing	*Bacteroides* species decrease and *Clostridium* species increase in the feces of mice that are exposed to chronic stress.	Bailey et al. [29]
**Adult male Sprague-Dawley rats**	Porsolt’s test, levels of components of the toll-like receptor 4 (TLR-4) signaling pathway, of lipopolysaccharide (LPS), and of different inflammatory, oxidative/nitrosative, and anti-inflammatory mediators were measured by RT-PCR, Western blot and/or ELISA in brain prefrontal cortex.	Plasma LPS, LPS-binding protein, brain LPS receptor and TLR4 levels were found to be high.	Garate et al. [30]
**871 European mothers and their children**	Maternal interview, The Strengths and Difficulties Questionnaire, Conners Rating Scales Revised, The Centre for Epidemiological Studies Depression Scale for Children, intelligence tests	Correlation between usage of antibiotics before the age of one year and adulthood depression.	Slykerman et al. [31]
**NIH Swiss mice**	Novel object recognition test, light/dark box test, acute restraint stress analysis, corticosterone immunoassay, DNA extraction, and high-throughput DNA sequencing	Long-term usage of broad-spectrum antibiotics change gut microbiota composition permanently.	Desbonnet et al. [32]
**Ninety-two patients with early stage schizophrenia**	Scale for the Assessment of Negative Symptoms (SANS), Positive and Negative Syndrome Scale (PANSS), Clinical Global Impression Scale (CGI), and cognitive tests.	Minocycline creates antipsychotic-like effect.	Liu et al. [33]
**112 depressed patients and 28 normal controls**	Hamilton Depression Rating Scale. Indirect ELISA method was employed to assay the immunoglobulin M (IgM) and IgA responses against the LPS of the commensal bacteria	Due to leaky gut, endotoxins originated from microorganisms join the systemic circulation.	Maes et al. [47]
**Female Wistar rats**	Partial restraint stress tests, oral administration of *Lactobacillus farciminis*	*Lactobacillus farciminis* suppressed stress-induced hyperpermeability, endotoxemia and prevented hypothalamic–pituitary–adrenal (HPA) axis stress response and neuroinflammation.	Ait-Belgnaoui et al. [48]
**Specific pathogen-free mice**	Open field test, real-time PCR analysis to assess neuroinflammation, HPLC for analysis of 5-HT and 5-HIAA in brain and intestine	In autistic mice, neuroinflammatory parameters increase.	de Theije et al. [50]
**Pregnant C57BL/6N mice**	Intestinal permeability assay, 16S rRNA gene sequence analysis, behavioral tests	Autistic behaviors can be eliminated by adding *Bacteroides fragilis* to the microbiota. *Bacteroides fragilis* can repair leaky gut.	Hsiao et al. [53]
**Male Wistar rats, healthy subjects**	Hopkins Symptom Checklist-90, Hospital Anxiety and Depression Scale, Perceived Stress Scale, Coping Checklist, urinary free cortisol levels	*Lactobacillus helveticus* and *Bifidobacteria longum* reduced the anxiety levels in rats and psychological distress in volunteers.	Messaoudi et al. [54]
**Germ-free mice**	Acute restraint test, maternal behavior observation	Brain-derived neurotrophic factor (BDNF), N-methyl D-aspartate (NMDA) 2a levels decreased in the hippocampus of germ-free mice.	Sudo et al. [63]
**Healthy volunteers**	Hospital Anxiety and Depression Scale, Hopkins Symptoms Checklist, urinary free cortisol levels	Anxiety levels recessed, and urine free cortisol levels decreased.	Messaoudi et al. [64]
**37 depressed patients, and 18 non-depressed controls**	Montgomery-Asberg Depression Rating Scale, Illumina deep sequencing of 16S rRNA gene amplicons	*Bacteroides* levels are high and *Lachnospiraceae* levels are low in the stool of depression patients.	Naseribafrouei et al. [72]
**Specific pathogen–free BALB/c mice, germ-free BALB/c mice**	Levels of serotonin, noradrenaline, dopamine, and brain-derived neurotropic factor were assessed by enzyme-linked immunosorbent assay	In germ-free mice BDNF levels and 5HT_1A_ expression are low.	Bercik et al. [80]
**Germ free mice, Specific pathogen-free mice**	Elevated plus maze	In germ-free mice anxiety levels are low.	Neufeld et al. [90]
**Germ free mice**	Open field test, elevated plus maze, light–dark box test	Microbial colonization affects brain development and behavior.	Diaz Heijtz et al. [92]
**Sprague–Dawley rat pups**	Forced swim test, corticosterone enzyme immunoassay, brain monoamine analysis, plasma tryptophan pathway analysis	*Bifidobacterium infantis* led to decreases in anxiety and depression levels and increases in central norepinephrine levels.	Desbonnet et al. [94]
**Healthy male participants**	Self-report stress measures, cognitive assessments, resting electroencephalography, plasma interleukin 10 (IL10), IL1β, IL6, IL8, and TNFα levels, whole blood toll-like receptor-4 agonist-induced cytokines, salivary cortisol analysis.	*Lactobacillus rhamnosus* was not found to be superior to placebo.	Kelly et al. [108]
**Patients with major depression and healthy subjects**	Hamilton’s Depression Scale, Montgomery–Asberg Depression Rating Scale, Serum tumor necrosis factor-a, IL-1b, IL-6, brain-derived neurotrophic factor analysis, polymerase chain reaction and pyrosequencing	Increases in the levels of *Alistipes, Enterobacteriaceae Bacteroides, Proteobacteria* and *Actinobacteria* and decreases in the levels of *Faecalibacterium* and *Firmicutes* in the fecal samples of depression patients.	Jiang et al. [114]
**Male germ-free Kunming mice and Specific pathogen-free Kunming mice**	Open-field test, Y-maze, tail suspension test, forced swimming test	After stool transplantation from depression patients to germ-free mice started to show depression-like behaviors.	Zheng et al. [116]
**Patients with major depression and healthy subjects, adult male Sprague-Dawley rats**	Plasma C-reactive protein, panel of cytokines, salivary cortisol levels, plasma tryptophan and kynurenine, plasma lipopolysaccharide binding protein, 16sRNA metagenomic sequencing for fecal samples	After fecal microbiota transplantation from depressed persons to rats, depression- and anxiety-like behaviors were observed in the laboratory animals.	Kelly et al. [117]
